# The impact of challenge and hindrance stressors on knowledge hiding: the mediating role of job crafting and work withdrawal

**DOI:** 10.3389/fpsyg.2024.1465480

**Published:** 2024-11-26

**Authors:** Le Wang, Yongtang Jia, Kun Xie

**Affiliations:** ^1^School of Education, Huazhong University of Science and Technology, Wuhan, China; ^2^School of Preschool Education, Yuzhang Normal University, Nanchang, China; ^3^School of Primary Education, Yuzhang Normal University, Nanchang, China

**Keywords:** challenge stressors, hindrance stressors, knowledge hiding, job crafting, work withdrawal, empowering leadership

## Abstract

The purpose of this study is to explore the impact of challenge and hindrance stressors on employees’ knowledge-hiding behavior, based on self-reported data from 493 Chinese preschool teachers. The findings indicate that both challenge and hindrance stressors significantly increase knowledge hiding, with hindrance stressors exerting a more pronounced effect. Furthermore, the study reveals the mediating roles of job crafting and work withdrawal, highlighting the distinct mechanisms involved with these stressors. Specifically, challenge stressors increase the likelihood of knowledge hiding through work withdrawal while simultaneously decreasing it through job crafting; notably, the former pathway has a greater effect. In contrast, hindrance stressors consistently exert detrimental effects, amplifying the probability of knowledge hiding through both mediators, which explains their stronger impact compared to challenge stressors. Additionally, empowering leadership plays a crucial moderating role in this relationship. The adverse influence of hindrance stressors on knowledge hiding, as mediated by job crafting, intensifies under high levels of empowering leadership. These findings not only validate the newly constructed parallel mediation model within an educational context but also provide practical strategies for kindergarten administrators regarding knowledge management. Such strategies include effectively distinguishing and managing different types of job stressors, enhancing skills to empower employees, and organizing regular knowledge-sharing activities.

## 1 Introduction

In the knowledge economy era, knowledge has become an essential strategic resource for organizations (Rhee and Choi, 2017). Therefore, fostering knowledge sharing is crucial for promoting organizational innovation and sustaining competitive advantages (Garg and Anand, 2020). Despite numerous efforts made by organizations to motivate employees to engage in knowledge-sharing behavior, the prevalence of knowledge-hiding behavior remains high (Connelly et al., 2019). Knowledge hiding refers to the intentional act of withholding requested information or providing non-requested information in response to a colleague’s inquiry. This behavior includes playing dumb (i.e., feigning ignorance of relevant information), evasive hiding (i.e., providing inaccurate information or a misleading promise of a complete answer in the future), and rationalized hiding (i.e., offering justifications for withholding the information). The defining characteristics of knowledge hiding are that the knowledge is requested and the act of hiding is intentional (Connelly et al., 2012). Although considered counterproductive behavior inhibiting employees’ innovation and undermining organizational performance (Hernaus et al., 2019), organizations cannot compel their members to cease engaging in knowledge hiding due to its value as a personal resource aiding individual success (Pereira and Mohiya, 2021). Consequently, knowledge sharing does not occur spontaneously. Previous studies have highlighted the significant influence of various variables—such as knowledge characteristics (e.g., knowledge complexity, refer to Chen, 2020), individual factors (e.g., self-efficacy, refer to Kumar Jha and Varkkey, 2018), and contextual factors (e.g., ethical leadership, refer to Belschak et al., 2018)—on knowledge-hiding behavior.

The challenge-hindrance stressor framework (CHSF) posits that, based on an individual’s assessment of their ability to cope with stressors, job stressors can be classified into challenge stressors and hindrance stressors (Cavanaugh et al., 2000). Challenge stressors refer to those that individuals can overcome, providing subsequent benefits. They have a motivating effect that helps employee’s complete tasks and promotes self-growth. Examples of challenge stressors include time pressure, high workload, and diverse responsibilities. Conversely, hindrance stressors, such as role ambiguity, job insecurity, bureaucracy, and red tape, are those that individuals cannot overcome. These stressors impede goal attainment and self-growth without offering any subsequent advantages. While there are fundamental distinctions between challenge and hindrance stressors, findings regarding their correlation with knowledge hiding are conflicting. Some studies indicate a positive correlation between both types of stressors and knowledge hiding (Zhang and Min, 2022), while others suggest that only hindrance stressors are positively correlated with knowledge hiding, with challenge stressors showing a negative correlation (Montani et al., 2024). Despite these inconsistent conclusions, the results imply that the influence of job stressors on knowledge-hiding behavior may operate through both motivation and depletion paths. The hypothesis of these two paths stems from the job demands-resources (JD-R) model proposed by Demerouti et al. (2001). According to the JD-R model, job characteristics can be categorized into job demands (e.g., physical workload and time pressure) and job resources (e.g., colleague assistance and performance feedback). Job demands exhaust employees’ energy, leading to negative outcomes like job burnout and low organizational commitment, illustrating the depletion path mechanism. Conversely, job resources facilitate task completion and lessen employees’ physical and mental strain, resulting in positive effects like work engagement and high organizational commitment, thereby displaying the motivation path mechanism. Notably, within this model, all types of job stressors are considered job demands that lead to negative outcomes.

Limited evidence exists regarding the impact of the distinct job stressors—challenge and hindrance—on employees’ knowledge-hiding behavior (Škerlavaj et al., 2023; Wang et al., 2024). Previous studies have primarily focused on specific stressors (e.g., time pressure, refer to Škerlavaj et al., 2018) or have only examined the positive or negative effects of certain types of stressors (Zhang and Min, 2022; Montani et al., 2024). Moreover, the conclusions of the existing studies are inconsistent. Therefore, the primary aim of this study is to investigate the influence of both challenge and hindrance stressors on employees’ knowledge-hiding behavior. Unlike the JD-R model, which considers job stressors solely as demands leading to negative outcomes, and the CHSF, which evaluates challenge stressors positively and hindrance stressors negatively, this study proposes a parallel mediation model that integrates both the CHSF and JD-R. Specifically, this model explores the underlying mechanisms through which both types of job stressors influence knowledge hiding via dual pathways of motivation and depletion. Furthermore, leaders play a crucial role within organizations, and their management style significantly affects employees’ attitudes and behaviors (Kim et al., 2018). Research confirms that leadership style has a substantial impact on employees’ evaluations of both the challenge and hindrance aspects of stressors (Arnold, 2017; She et al., 2024). Therefore, it is reasonable to incorporate leadership style as a moderating variable within the theoretical model.

Regarding mediating variables, significant correlations have been identified between challenge stressors and job crafting (Chen et al., 2021), as well as between hindrance stressors and work withdrawal (Piccoli et al., 2019). Therefore, job crafting and work withdrawal are considered mediating variables representing the motivation path and depletion path, respectively. This approach enhances our understanding of the relationships among job crafting, work withdrawal, and knowledge hiding while addressing the current lack of evidence in this area. Furthermore, in terms of the moderating variable, this study selects empowering leadership from various leadership styles. This choice is based on its sensitivity to resource ownership and its dual potential to act as either a beneficial resource or a detrimental burden, depending on the availability of personal resources (Wang and Sun, 2019). Thus, it functions as a double-edged sword (Cheong et al., 2016). Given the differing impacts of challenge stressors and hindrance stressors on employees’ ownership of personal resources (Cavanaugh et al., 2000), the interaction terms between empowering leadership and these two types of stressors may display varying moderating effects on the mediating pathways. This exploration contributes to a clearer understanding of the boundary conditions under which empowering leadership exerts distinct influences, thereby addressing ongoing controversies surrounding this topic (Wang and Sun, 2019).

Notably, this study focuses on preschool teachers who encounter significant job stressors (Nislin et al., 2015; Zheng et al., 2020). The typical stressors faced by Chinese preschool teachers include heavy workloads due to large class sizes, substantial responsibilities for ensuring children’s safety, bureaucratic challenges in kindergarten management, and a lack of support for career development (Zheng et al., 2020). It is evident that preschool teachers are exposed to both challenge and hindrance stressors. Additionally, as a highly specialized profession, knowledge sharing is crucial for the development of teachers, students, and schools alike (Li, 2021). Studies conducted in various regions of China, utilizing cluster sampling methods, have revealed that preschool teachers experience a significant issue regarding limited professional knowledge. Over 30% of these teachers have less than 3 years of teaching experience, and approximately 15% lack a professional background in preschool education (Huang et al., 2024; Qiu et al., 2020). This situation underscores the urgent need for enhanced knowledge sharing among these educators. The dual dilemma of job stressors and knowledge hiding among Chinese preschool teachers makes them a representative sample of this study. Thus, this study investigates how challenge and hindrance stressors impact preschool teachers’ knowledge-hiding behavior. It examines the mediating roles of job crafting and work withdrawal while also exploring the moderating role of empowering leadership. The aim is to uncover the underlying mechanisms within an educational context and provide practical suggestions for kindergarten administrators to implement effective knowledge management strategies.

## 2 Research hypotheses and model construction

### 2.1 Challenge-hindrance stressors and knowledge hiding

Although the CHSF acknowledges the clear distinctions between challenge stressors and hindrance stressors, their impact on knowledge hiding could be similarly positive. According to the COR theory, resources are defined as “anything perceived by the individual to help attain his or her goals” (Halbesleben and Wheeler, 2015). Tenure, seniority, time, knowledge, and social support are all valuable resources for individuals. Intrinsic motivation drives individuals to acquire, conserve, and maintain resources throughout their lives. When exposed to challenge or hindrance stressors, individuals perceive the need to consume additional personal resources to cope with job demands. This creates a significant threat to their existing resources. Notably, individuals are more sensitive to resource loss than to resource acquisition (Hobfoll et al., 2018). The impact of resource loss is both rapid and persistent. The perception of a potential threat to resource loss often results in considerable strain and emotional exhaustion (Lepine et al., 2005). Consequently, individuals tend to adopt primacy-of-loss coping behaviors. These behaviors aim to minimize resource expenditure, thus alleviating the speed and extent of resource depletion. Therefore, when confronted with knowledge requests from peers, individuals may choose to hide knowledge as a valuable resource for several reasons. First, knowledge hiding serves as a means to avoid immediate depletion of time, energy, and other personal resources. Second, it helps prevent potential loss of personal resources, such as competitive advantages and opportunities for job promotion. Therefore, we hypothesize that:

H1a: Challenge stressors have a positive impact on knowledge hiding.

H2b: Hindrance stressors have a positive impact on knowledge hiding.

### 2.2 Mediating role of job crafting

Job crafting refers to the process through which employees proactively redesign their work tasks, cognitive perceptions, and social relationships to better align with their personal values, preferences, abilities, and motivations. These autonomous behaviors allow individuals to redefine the purpose and meaning of their work (Wrzesniewski and Dutton, 2001). Job crafting encompasses three dimensions: task crafting (i.e., involving changes to the order, quantity, and scope of work tasks), cognitive crafting (i.e., enhancing one’s perception of work itself), and relational crafting (i.e., improving social relationships within the workplace).

Distinct from knowledge hiding, the impacts of challenge and hindrance stressors on employees’ job crafting are fundamentally different. Challenge stressors, on one hand, are perceived by employees as opportunities for self-growth and career advancement (Lepine et al., 2005). They motivate individuals to deliver outstanding performance to obtain resources in return. An optimistic anticipation of resource rewards fosters positive work attitudes and behaviors, making job crafting identified as one of the most effective coping strategies. Through task crafting, employees can independently redesign work tasks, allowing them to allocate personal resources more efficiently and cope better with challenge stressors. Similarly, cognitive crafting enables employees to view work meaning in a more positive light, helping them recognize the inherent challenges and opportunities present in their roles. Finally, relationship crafting improves the quality, scope, and frequency of interpersonal interactions with colleagues, thereby creating a robust interpersonal network that facilitates greater resource support from peers (Wrzesniewski and Dutton, 2001). On the contrary, hindrance stressors are perceived by employees as detrimental factors that exacerbate the depletion of personal resources. Pessimistic expectations regarding resource depletion hinder positive attitudes and behaviors among employees, including job crafting. When faced with hindrance stressors, individuals are required to invest significant personal resources to cope with them. This requirement can lead to increased resource depletion if individuals must allocate additional resources to job crafting. Consequently, those who are highly sensitive to resource loss (Hobfoll et al., 2018) typically refrain from job crafting. Instead, they may opt to minimize their resource investment in work endeavors as a strategy of mitigating potential losses.

According to the COR theory, individuals’ initial access to resources also facilitates further resource acquisition, resulting in what is known as resource gain spirals (Halbesleben and Wheeler, 2015). Once employees achieve resource rewards through job crafting, they enter a motivation pathway that encourages further investment behavior. In response to knowledge requests from colleagues, individuals often engage in knowledge sharing as a form of resource investment. The strong interpersonal networks developed through relationship crafting promote mutual assistance among employees (Kira et al., 2010), particularly regarding knowledge sharing. Additionally, the enhanced sense of meaning at work derived from cognitive crafting encourages employees to share knowledge for the benefit of the organization (Ghitulescu, 2007). Furthermore, improvements in job performance and self-efficacy resulting from task crafting alleviate concerns about potential loss of personal resources (Chatman, 1989). This, in turn, lessens the likelihood of knowledge hiding as a strategy for resource protection. Conversely, hindrance stressors interfere with employees’ job crafting, leading to a loss of motivation for further resource-investment behavior and an increased tendency to engaging in knowledge hiding. Therefore, we hypothesize that:

H2a: Job crafting mediates the relationship between challenge stressors and knowledge hiding.

H2b: Job crafting mediates the relationship between hindrance stressors and knowledge hiding.

### 2.3 Mediating role of work withdrawal

Work withdrawal refers to a series of covert retaliatory actions taken by employees who perceive unfavorable circumstances within their organization. These actions aim to disengage from work tasks and weaken connections with colleagues (Gupta and Jenkins, 1991). Work withdrawal can be categorized into two dimensions: psychological withdrawal behavior and physical withdrawal behavior (Lehman and Simpson, 1992).

In contrast to job crafting, challenge and hindrances stressors are likely to have a consistent impact on work withdrawal. Both types of stressors can inevitably endanger an individual’s available resources (Rodell and Judge, 2009), triggering a series of work withdrawal behaviors among employees (Schaubroeck et al., 1989). On the one hand, employees may exhibit psychological withdrawal behavior, such as inattention and negative work attitudes. On the other hand, they may engage in physical withdrawal behavior, displaying more serious avoidance actions like tardiness, early departure, and absence. These work disengagement behaviors effectively reduce resource depletion for employees. It is noteworthy that, compared to the straightforward adverse impact caused by hindrance stressors, challenge stressors result in more intricate outcomes. This effect is characterized by a “double-edged sword” phenomenon that yields both beneficial and detrimental outcomes (Webster et al., 2010; Lepine et al., 2004).

According to COR theory, when resources are continuously lost without effective compensation, individuals enter resource loss spirals. This means that an initial loss of resources leads to further losses, potentially resulting in resource desperation (Demerouti and Bulters, 2004). Although employees attempt to reduce resource loss through work withdrawal, this strategy merely decelerates rather than eradicates such loss through resource recovery. Therefore, once employees choose work withdrawal as an adaptive coping behavior, they enter a depletion path that reduces their willingness to invest further in resources. Consequently, when faced with knowledge requests from colleagues, individuals are more likely to opt for knowledge hiding for the following reasons. Firstly, work withdrawal by employees engenders dissatisfaction among leaders and colleagues due to task avoidance and decreased performance. This dissatisfaction increases the impetus behind knowledge hiding through heightened interpersonal conflict. Secondly, work withdrawal causes employees to become unfamiliar with job content while their professional competence declines and their initiative for self-improvement diminishes. This decline reduces their ability to share knowledge (Afrahi et al., 2022). Lastly, work withdrawal serves as a response to discontentment with the organizational working environment. In this context, knowledge hiding may be perceived as a covert retaliatory behavior against the organization (Gupta and Jenkins, 1991). Therefore, we hypothesize that:

H3a: Work withdrawal mediates the relationship between challenge stressors and knowledge hiding.

H3b: Work withdrawal mediates the relationship between hindrance stressors and knowledge hiding.

### 2.4 Moderating role of empowering leadership

Empowering leadership encompasses a variety of behaviors and processes that leaders use to empower employees. These include delegating authority, involving employees in decision-making, sharing information, and assigning responsibility (Konczak et al., 2000). It can be considered a form of “super leadership,” (Manz and Sims, 1991) designed to enhance employees’ sense of the meaningfulness of work, as well as their self-efficacy, work autonomy, and influence at work (Ahearne et al., 2005).

However, contrary to early findings, an increasing body of evidence supports the double-edged sword effect of empowering leadership (Cheong et al., 2016). It can serve as both a valuable resource (Amundsen and Martinsen, 2014) and an excessive burden (Wong and Giessner, 2018). A crucial boundary condition that determines the specific effects of empowering leadership is the individual’s status regarding resource ownership. When resources are abundant, empowering leadership tends to yield beneficial effects more easily. Conversely, in resource-scarce situations, it is more likely to result in detrimental consequences (Wang and Sun, 2019). Challenge stressors can effectively enhance employees’ work motivation, improve job performance, and generate resource rewards (Cavanaugh et al., 2000). Consequently, empowering leadership is more likely to function as a powerful resource when employees manage these stressors. According to the “coping” hypothesis in the JD-R model, the positive impact of high-level job resources is more pronounced in situations characterized by high-level job demands (Lewig et al., 2007). Moreover, the “buffering” hypothesis suggests that work-related resources can help mitigate the adverse effects of resource depletion (Bakker et al., 2005). In environments where ample resources are provided through highly empowering leadership, challenge stressors are more likely to inspire employees to engage in job crafting. This, in turn, reduces their inclination toward work withdrawal and ultimately decreases instances of knowledge hiding. Specifically, a leader’s empowering behavior builds trust in subordinates’ capabilities and performance. This enhances their self-efficacy (Konczak et al., 2000) while fostering psychological security (Srivastava et al., 2006). Additionally, it cultivates a more inclusive working environment, which helps alleviate cognitive and emotional resource depletion (Harris et al., 2014). Empowering leaders also provide subordinates with greater autonomy, stimulating their motivation for job crafting (Wrzesniewski and Dutton, 2001). Conversely, less empowering leadership diminishes the support and resources available to employees, restricts their ability to engage in job crafting, and fosters feelings of insecurity. Ultimately, this results in disengagement from work and a higher incidence of knowledge-hiding behavior. Therefore, we hypothesize that:

H4a: Empowering leadership moderates the indirect relationship between challenge stressors and knowledge hiding through job crafting, such that this indirect relationship is stronger when empowering leadership is high.

H4b: Empowering leadership moderates the indirect relationship between challenge stressors and knowledge hiding through work withdrawal, such that this indirect relationship is weaker when empowering leadership is high.

Considering that hindrance stressors lead to continual resource depletion for employee’s striving to complete their tasks (Cavanaugh et al., 2000), individuals often find themselves in resource-deficient situations. Consequently, empowering leadership can become an excessive burden for employees facing hindrance stressors, negatively affecting their well-being and work performance (Lee et al., 2017). In environments where empowering leadership is prevalent, the adverse impacts of hindrance stressors on employees’ job crafting and work withdrawal are significantly amplified, ultimately increasing instances of knowledge-hiding behavior. Firstly, the self-leadership and self-management encouraged by empowering leadership can increase task complexity (Magni and Maruping, 2013). This may lead to role stress (Humborstad et al., 2014) and further drain employees’ resources. Moreover, employees may misinterpret empowering behaviors from leaders as a lack of responsibility (Hinkin and Schriesheim, 2008), or underestimate the importance of certain tasks (Lee et al., 2017), leading to decreased work motivation (Skogstad et al., 2014). As a result, highly empowering leadership can induce stress responses in employees (Cheong et al., 2016), diminishing their engagement in job crafting while increasing occurrences of work withdrawal and knowledge hiding. Conversely, less empowering leadership can alleviate resource depletion caused by task complexity and extra-role behaviors at work. This approach ultimately reduces resource depletion among employees. Additionally, it encourages greater job crafting, decreases tendencies for work withdrawal, and diminishes instances of knowledge-hiding behavior. Therefore, we hypothesize that:

H4c: Empowering leadership moderates the indirect relationship between hindrance stressors and knowledge hiding through job crafting, such that this indirect relationship is stronger when empowering leadership is high.

H4d: Empowering leadership moderates the indirect relationship between hindrance stressors and knowledge hiding through work withdrawal, such that this indirect relationship is stronger when empowering leadership is high.

**Figure 1:**
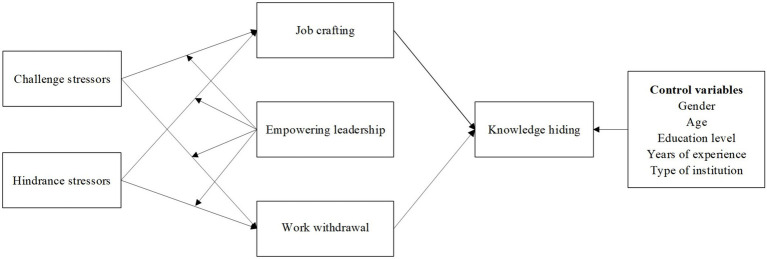
Theoretical model of the present study.

As such, the theoretical model of the present study is illustrated in Figure 1.

## 3 Materials and methods

### 3.1 Participants and procedure

The principals of the kindergartens disseminated the information regarding the open recruitment of volunteers for this study to the preschool teachers’ WeChat work groups. The information included details about the research purpose, research procedure, the principle of voluntary participation, and assurance of anonymity and confidentiality. Questionnaires were distributed through the Wenjuanxing online platform.^1^ Preschool teachers who wished to participate in this study could scan the QR code generated by Wenjuanxing on WeChat to complete the questionnaires on their mobile phones.

To address the potential issue of common method bias and the risk of invalid responses caused by respondent fatigue, this study adopted a three-time data collection method. Data were collected at one-month intervals, consistent with the methodologies used by Tong et al. (2021) and Qiu et al. (2023). During time 1, challenge stressors, hindrance stressors, and empowering leadership were measured, resulting in obtaining 574 questionnaires. During time 2, job crafting and work withdrawal were measured, resulting in obtaining 569 questionnaires. During time 3, knowledge hiding was measured while demographic information was collected, resulting in obtaining 551 questionnaires. Several participants withdrew during the process due to resignation or personal reasons. Ultimately, a total of 493 valid questionnaires were obtained by matching the data from the three time points using the last six digits of phone numbers and filtering out invalid responses. The final sample primarily consists of females (97.4%). Most of them were under 34 years old (85.0%) and held either college degrees (46.5%) or undergraduate degrees (47.9%). Additionally, over half of the participants work in public kindergartens (59.4%) and have less than 6 years of experience (66.7%). Sample characteristics are presented in Table 1.

**Table 1:** Sample descriptive statistics (*N* = 493).

Characteristic	Category	Percentage
Gender	Male	2.6%
	Female	97.4%
Age	≤25	30.4%
	26–34	54.6%
	35–44	9.7%
	≥45	5.3%
Education level	Junior high school and below	0.8%
	High school	4.7%
	Junior college	46.5%
	Undergraduate	47.9%
	Master or above	0.2%
Years of experience	≤3	30.8%
	4–6	35.9%
	7–10	14.2%
	11–14	10.1%
	≥15	8.9%
Type of institution	Public kindergarten	59.4%
	Private kindergarten	40.6%

Additionally, results from a one-sample T-test indicated that there were no significant differences in age (*t* = 0.86, *p* > 0.05), gender (*t* = 0.39, *p* > 0.05), education level (*t* = −0.65, *p* > 0.05), years of experience (*t* = 0.77, *p* > 0.05), and type of institution (*t* = 1.58, *p* > 0.05) between valid samples (*N* = 493) and invalid samples (*N* = 81). This suggests that the loss of samples did not significantly impact the representativeness of the data.

### 3.2 Measures

All measures were assessed using a five-point Likert scale, ranging from 1 (indicating strong disagreement) to 5 (indicating strong agreement).

Challenge and hindrance stressors were measured with the 11-item scale from Cavanaugh et al. (2000). An example item was “The amount of time I spend at work is significant.” The Cronbach’s *α* coefficient of the scale was 0.861.

Knowledge hiding was assessed using the 12-item scale from Connelly et al. (2012). An example item was “I pretended that I did not know the information.” The Cronbach’s α coefficient of the scale was 0.896.

Job crafting was evaluated with the 15-item scale developed by Slemp and Vella-Brodrick (2013). An example item was “I make an effort to get to know people well at work.” The Cronbach’s α coefficient of the scale was 0.938.

Work withdrawal was measured using the 12-item scale from Lehman and Simpson (1992). An example item was “I left work early without permission.” The Cronbach’s α coefficient of the scale was 0.968.

Empowering leadership was assessed with the 10-item scale developed by Ahearne et al. (2005). An example item was “My manager allows me to do my job my way.” The Cronbach’s α coefficient of the scale was 0.963.

### 3.3 Data analysis

Confirmatory factor analysis was conducted using Amos 26.0. Various statistical analyses were performed using SPSS 25.0, including one-sample T-test, common method bias tests, multicollinearity diagnostics, descriptive analysis, correlation analysis, hierarchical regression analysis, and one-way ANOVA. To explore the mediating and the moderated mediating effects, Models 4 and 7 of the SPSS macro program PROCESS 4.1 developed by Hayes (2017) were applied. A 95% confidence interval (CI) and 5,000 bootstraps were utilized for these analyses. Additionally, if a statistically significant interaction term was identified, simple slope analyses using the pick-a-point method were conducted to illustrate the moderating role.

## 4 Results

### 4.1 Common method bias test and multicollinearity diagnostics

To address potential common method bias, this study utilized both program control and statistical control methods. Program control involved collecting questionnaire data at three different time points, while statistical control included conducting the Harman single-factor test and confirmatory factor analysis. The results of the Harman single-factor test showed the extraction of nine factors from the principal component analysis, accounting for 74.543% of the total variance. However, Factor 1 only explained 32.273% of the total variance, which was significantly below the critical threshold of 40%. Additionally, the results of the confirmatory factor analysis (refer to Table 2) indicated that, among the various models considered, including alternative factor models, the six-factor model demonstrated superior fit indices (χ^2^/df = 2.758; CFI = 0.904; TLI = 0.900; IFI = 0.905; RMSEA = 0.060). These findings suggest that significant common method bias was not present in this study.

**Table 2:** Results of confirmatory factor analysis (CFA).

Model	χ^2^	df	χ^2^/df	IFI	TLI	CFI	RMSEA
Six factors: CS; HS; EL; JC; WW; KH	4675.368	1,695	2.758	0.905	0.900	0.904	0.060
Five factors: CS + HS; EL; JC; WW; KH	5301.307	1700	3.118	0.885	0.880	0.885	0.066
Four factors: CS + HS + EL; JC; WW; KH	6906.572	1704	4.053	0.834	0.827	0.833	0.079
Three factors: CS + HS + EL; JC + WW; KH	12787.992	1707	7.492	0.645	0.632	0.645	0.115
Two factors: CS + HS + EL; JC + WW + KH	18355.900	1709	10.741	0.467	0.447	0.466	0.141
One factor: CS + HS + EL + JC + WW + KH	22732.839	1710	13.294	0.327	0.302	0.326	0.158

CS, Challenge stressors; HS, Hindrance stressors; EL, Empowering leadership; JC, Job crafting; WW, Work withdrawal; and KH, Knowledge hiding.

To assess potential multicollinearity, this study employed the variance inflation factor (VIF). The results indicated that the VIF values for all predictor variables within the regression models ranged from 1.043 to 2.196, all of which fell below the threshold of 3. Additionally, the tolerance values varied from 0.455 to 0.958, exceeding the minimum acceptable threshold of 0.1. These findings suggest that multicollinearity was not a significant issue within the regression models.

### 4.2 Descriptive statistics and correlation analysis

The results of the correlation analysis (refer to Table 3) demonstrated significant positive correlations between challenge stressors and both job crafting (*r* = 0.156, *p* < 0.01) and work withdrawal (*r* = 0.277, *p* < 0.01). Conversely, hindrance stressors exhibited a negative correlation with job crafting (*r* = −0.383, *p* < 0.01) and a positive correlation with work withdrawal (*r* = 0.586, *p* < 0.01). Additionally, a significant negative correlation was found between job crafting and knowledge hiding (*r* = −0.420, *p* < 0.01). Moreover, a significant positive correlation existed between work withdrawal and knowledge hiding (*r* = 0.674, *p* < 0.01), providing initial support for the hypotheses.

**Table 3:** Means, standard deviations (SD), and intercorrelations among the main variables.

Variable	1	2	3	4	5	6	7	8	9	10	11
1. Gender	1										
2. Age	−0.022	1									
3. Education level	−0.061	−0.121**	1								
4. Years of experience	0.003	0.687**	0.044	1							
5. Type of institution	0.049	0.044	−0.194**	−0.047	1						
6. Challenge stressors	−0.062	0.154**	0.095*	0.152**	−0.095*	1					
7. Hindrance stressors	−0.061	−0.107*	0.125**	−0.082	−0.066	0.204**	1				
8. Empowering leadership	0.057	0.191**	−0.147**	0.126**	0.049	0.086	−0.394**	1			
9. Job crafting	0.044	0.296**	−0.105*	0.230**	0.048	0.156**	−0.383**	0.707**	1		
10. Work withdrawal	−0.079	−0.209**	0.159**	−0.143**	−0.094*	0.277**	0.586**	−0.337**	−0.366**	1	
11. Knowledge hiding	−0.130**	−0.155**	0.175**	−0.077	−0.153**	0.171**	0.476**	−0.306**	−0.420**	0.674**	1
Mean	1.972	1.899	3.420	2.304	1.428	4.226	2.777	3.807	3.815	2.952	2.981
SD	0.166	0.777	0.625	1.253	0.495	0.689	0.909	0.710	0.534	0.974	0.615

*p < 0.05; **p < 0.01.

Furthermore, gender, age, education level, years of experience, and type of institution were found to have significant correlations with several focal variables. Therefore, they were included as control variables in the subsequent data analysis.

### 4.3 Hypothesis testing

#### 4.3.1 Tests of main effect and mediating effect

The results of the hierarchical regression analysis (refer to Table 4) revealed significant positive impacts of both challenge stressors (M9, *β* = 0.151, *p* < 0.01) and hindrance stressors (M11, *β* = 0.300, *p* < 0.01) on knowledge hiding. Notably, hindrance stressors demonstrated a stronger influence, thus supporting both H1a and H1b.

**Table 4:** Results of the hierarchical regression analysis.

	Job crafting				Work withdrawal				Knowledge hiding			
	M1	M2	M3	M4	M5	M6	M7	M8	M9	M10	M11	M12
Control variables												
Gender	0.162	0.044	0.082	0.018	−0.334	−0.222	−0.248	−0.231	−0.411*	−0.249*	−0.355*	−0.250*
Age	0.153**	0.084**	0.150**	0.087**	−0.259**	−0.195**	−0.169**	−0.152*	−0.131**	0.001	−0.093*	−0.001
Education level	−0.071	0.002	−0.029	0.019	0.150*	0.085	0.098	0.086	0.111*	0.038	0.078	0.038
Years of experience	0.027	0.022	0.023	0.018	−0.041	−0.037	−0.007	−0.007	0.001	0.022	0.016	0.023
Type of institution	0.037	0.022	0.011	0.012	−0.074	−0.055	−0.074	−0.073	−0.127*	−0.091*	−0.121*	−0.093*
Independent variables												
Challenge stressors	0.098**	0.157			0.425**	0.745**			0.151**	0.019		
Hindrance stressors			−0.205**	0.164*			0.598**	0.554**			0.300**	0.047
Mediators												
Job crafting										−0.244**		−0.221**
Work withdrawal										0.366**		0.348**
Moderators												
Empowering leadership		0.615**		0.659**		−0.112		−0.134				
Interaction terms												
CS*EL		−0.026				−0.076						
HS*EL				−0.059**				0.002				
*R* ^2^	0.113	0.301	0.216	0.548	0.157	0.254	0.374	0.381	0.104	0.506	0.268	0.509
Δ*R*^2^	0.102	0.290	0.206	0.540	0.147	0.241	0.366	0.370	0.093	0.498	0.259	0.500
*F*	8.392***	26.071***	22.258***	73,333***	15.119***	20.569***	48.294***	37.181***	9.415***	61.953***	29.712***	62.604***

*p < 0.05; **p < 0.01; ***p < 0.001.

Furthermore, it was found that challenge stressors positively influenced job crafting (M1, *β* = 0.098, *p* < 0.01) and work withdrawal (M5, *b* = 0.425, *p* < 0.01). From M10, it was evident that job crafting (*β* = −0.244, *p* < 0.01) negatively impacted knowledge hiding, while work withdrawal (*β* = 0.366, *p* < 0.01) positively affected knowledge hiding. In contrast, hindrance stressors were associated with a negative effect on job crafting (M3, *β* = −0.205, *p* < 0.01) and a positive effect on work withdrawal (M7, *β* = 0.598, *p* < 0.01). Again, from M12, it was evident that job crafting (*β* = −0.221, *p* < 0.01) negatively impacted knowledge hiding, while work withdrawal (*β* = 0.348, *p* < 0.01) positively affected knowledge hiding. Therefore, job crafting and work withdrawal were identified as mediators in the relationship between challenge and hindrance stressors with knowledge hiding.

In addition, a mediation effect analysis was conducted using Model 4 in PROCESS 4.1 (Hayes, 2017), with a sample size of 5,000 and a 95% confidence interval. Tables 5, 6 revealed that the 95% confidence intervals for all direct paths include 0. In contrast, the 95% confidence intervals for all mediated paths exclude 0, indicating a significant perfect mediation effect. Specifically, the indirect effect value for the path “challenge stressors → job crafting → knowledge hiding” was −0.024 (95% CI = [−0.049, −0.004]). In comparison, the value for “challenge stressors → work withdrawal → knowledge hiding” was 0.155 (95% CI = [0.107, 0.209]), with the latter mediation path showing a significantly greater effect. Moreover, the indirect effect value for “hindrance stressors → job crafting → knowledge hiding” was 0.045 (95% CI = [0.023, 0.072]). For “hindrance stressors → work withdrawal → knowledge hiding,” it was 0.208 (95% CI = [0.164, 0.254]), again indicating that the latter mediation path had a significantly higher impact. These results further underscored the significant mediating roles of both job crafting and work withdrawal in the relationships between challenge and hindrance stressors with knowledge hiding, supporting H2a, H2b, H3a, and H3b.

**Table 5:** Results of mediation effect test (independent variable = challenge stressors).

	Effect	SE	95% CI
Direct effect			
Challenge stressors→Knowledge hiding	0.019	0.032	[−0.043, 0.082]
Indirect effect			
Challenge stressors→Job crafting→Knowledge hiding	−0.024	0.012	[−0.049, −0.004]
Challenge stressors→Work withdrawal→Knowledge hiding	0.155	0.026	[0.107, 0.209]
Indirect effect contrast			
Job crafting minus Work withdrawal	−0.179	0.026	[−0.232, −0.132]
Total indirect effect	0.131	0.031	[0.073, 0.194]
Total effect	0.151	0.039	[0.073, 0.228]

**Table 6:** Results of mediation effect test (independent variable = hindrance stressors).

	Effect	SE	95% CI
Direct effect			
Hindrance stressors→Knowledge hiding	0.047	0.027	[−0.007, 0.101]
Indirect effect			
Hindrance stressors→Job crafting→Knowledge hiding	0.045	0.013	[0.023, 0.072]
Hindrance stressors→Work withdrawal→Knowledge hiding	0.208	0.023	[0.164, 0.254]
Indirect effect contrast			
Job crafting minus Work withdrawal	−0.163	0.024	[−0.211, −0.115]
Total indirect effect	0.253	0.029	[0.198, 0.310]
Total effect	0.300	0.027	[0.248, 0.353]

#### 4.3.2 Tests of moderating effect

Based on the results from the hierarchical regression analysis presented in Table 4, the interaction term between challenge stressors and empowering leadership did not exhibit a significant moderating effect on job crafting (M2, *β* = −0.026, n.s.) or work withdrawal (M6, *β* = −0.076, n.s.). Similarly, the interaction term between hindrance stressors and empowering leadership did not show a significant moderating effect on work withdrawal (M8, *β* = 0.002, n.s.). Therefore, none of H4a, H4b, or H4d were supported.

**Figure 2:**
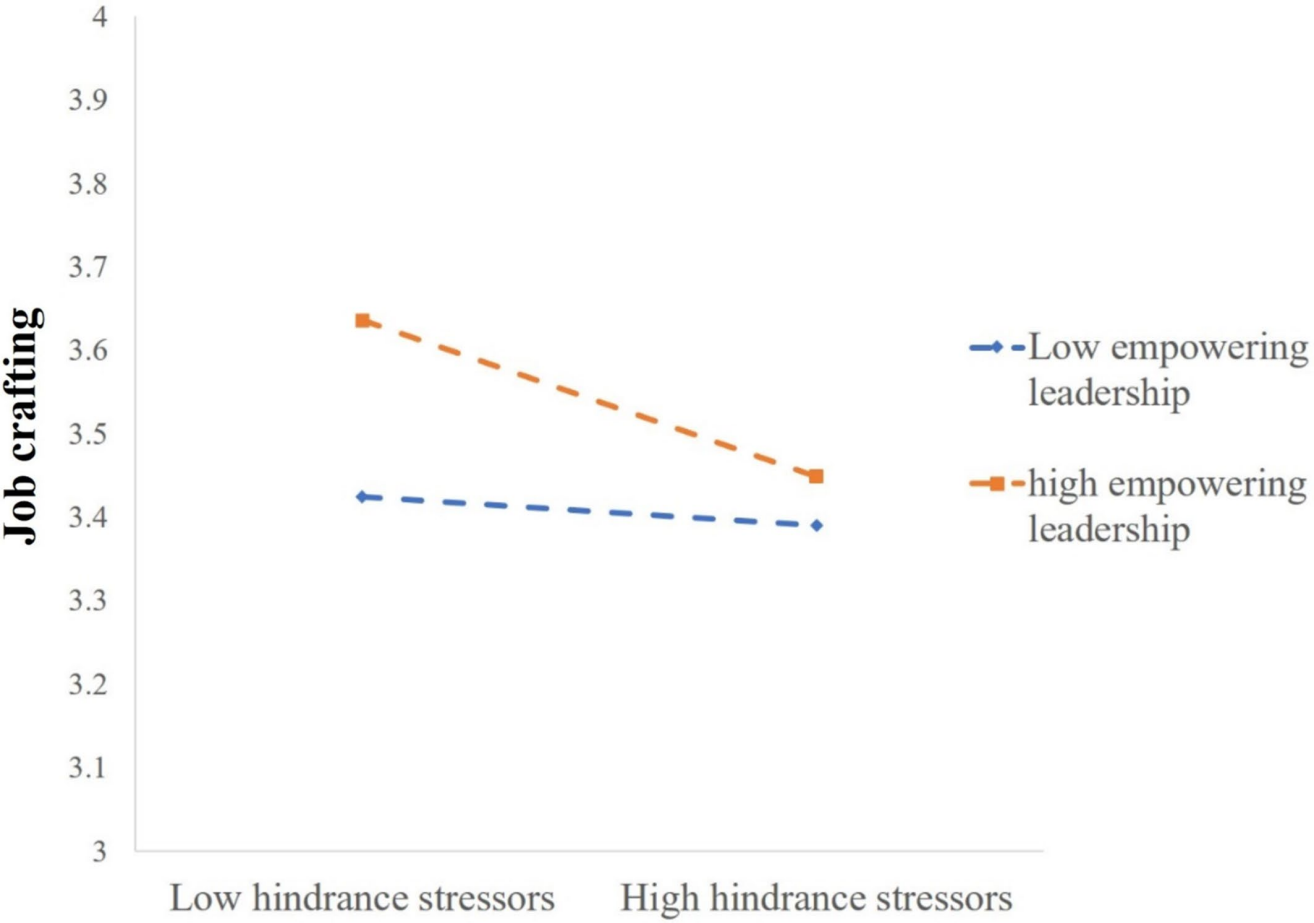
Moderating impact of empowering leadership on hindrance stressors and job crafting.

However, the interaction term between hindrance stressors and empowering leadership demonstrated a notable negative moderating effect on job crafting (M4, *β* = −0.059, *p* < 0.01). Furthermore, a moderated mediation effect test was conducted using Model 7 in PROCESS 4.1, with a sample size of 5,000 and a 95% confidence interval. The results (refer to Table 7) indicated that the indirect effect of job crafting between hindrance stressors and knowledge hiding was significant in the high empowering leadership group (βsimple = 0.023, 95% CI = [0.009, 0.042], excludes 0). In contrast, it was not significant in the low empowering leadership group (βsimple = 0.004, 95% CI = [−0.015, 0.024], includes 0). A simple slope plot is depicted in Figure 2. Hence, H4c was supported.

**Table 7:** Results of moderated mediation effect test.

Mediating path	Moderator	Effect	SE	95% CI
Hindrance stressors→Job crafting→Knowledge hiding	Empowering leadership low (-1SD)	0.004	0.010	[−0.015, 0.024]
	Empowering leadership mean	0.014	0.006	[0.003, 0.028]
	Empowering leadership high (+1SD)	0.023	0.009	[0.009, 0.042]

### 4.4 Supplementary analysis

To further clarify the impact of challenge stressors and hindrance stressors on focal variables, a one-way ANOVA analysis was conducted to compare group differences. Following Kelley’s (1939) criterion of the top 27% and bottom 27%, challenge stressors and hindrance stressors were categorized into high and low groups based on the reported scores. Subsequently, the results from each group were matched, resulting in the formation of four distinct groups: low challenge stressors—low hindrance stressors (*N* = 53), high challenge stressors—low hindrance stressors (*N* = 41), low challenge stressors—high hindrance stressors (*N* = 20), and high challenge stressors—high hindrance stressors (*N* = 59). The results of one-way ANOVA (refer to Table 8) revealed significant differences among groups in job crafting, work withdrawal, and knowledge hiding (*F* = 21.686; *F* = 45.547; *F* = 26.481; all *p* < 0.001).

**Table 8:** Results of one-way ANOVA (*N* = 173).

Groups	Job crafting		Work withdrawal		Knowledge hiding	
	M	SD	M	SD	M	SD
Low CSLow HS (*N* = 53)	3.97	0.48	2.14	0.73	2.57	0.57
High CSLow HS (*N* = 41)	4.37	0.44	2.46	1.07	2.63	0.66
Low CSHigh HS (*N* = 20)	3.37	0.42	3.32	0.93	3.19	0.38
High CSHigh HS (*N* = 59)	3.67	0.65	3.84	0.68	3.42	0.55
*F*	21.686***		45.547***		26.481***	
*Post hoc* multiple comparisons	**Dependent variable = Job crafting (Tamhane’s T2)**					
	High CSLow HS>Low CSLow HS>Low CSHigh HS & High CSHigh HS					
	**Dependent variable = Work withdrawal (Tamhane’s T2)**					
	Low CSHigh HS & High CSHigh HS>Low CSLow HS & High CSLow HS					
	**Dependent variable = Knowledge hiding (LSD)**					
	Low CSHigh HS & High CSHigh HS>Low CSLow HS & High CSLow HS					

CS, Challenge stressors; HS, Hindrance stressors. *p < 0.05; **p < 0.01; ***p < 0.001.

*Post hoc* multiple comparisons indicated that both the groups with low challenge stressors and high hindrance stressors, as well as the groups with high challenge stressors and high hindrance stressors, had the lowest scores in job crafting and the highest scores in work withdrawal and knowledge hiding. This suggests that employees facing high hindrance stressors are more likely to exhibit significantly decreased levels of job crafting, and increased tendencies for work withdrawal and knowledge hiding, regardless of their levels of challenge stressors.

## 5 Conclusion and discussion

### 5.1 Discussion

In accordance with the findings of Zhang and Min (2022), this study confirms the significant positive predictive impact of challenge stressors and hindrance stressors on knowledge-hiding behavior. Specifically, it has been found that a greater presence of these stressors among preschool teachers increase the likelihood of engaging in knowledge-hiding behavior. This outcome can be logically explained by COR theory. Since both types of stressors pose a threat to personal resources, employees tend to adopt the primacy-of-loss strategy of knowledge hiding to prevent resource depletion, which can ultimately lead to resource desperation (Hobfoll et al., 2018; Lepine et al., 2005).

#### 5.1.1 The mediating role of job crafting and work withdrawal

Although both types of stressors have a significant positive effect on knowledge-hiding behavior, the impact of hindrance stressors is stronger. This difference can be attributed to the mediating roles of job crafting and work withdrawal. While both job crafting and work withdrawal serve as mediators between the two types of stressors and knowledge hiding, their internal mechanisms differ. Challenge stressors enhance employees’ job crafting, increasing their motivation and capacity to share knowledge (i.e., motivation path). However, they also lead to greater work withdrawal among employees, which in turn heightens the likelihood of knowledge-hiding behavior (i.e., depletion path). Despite the stronger influence of the depletion path, the motivation path somewhat mitigates the positive impact of challenge stressors on knowledge hiding. This study further confirms the “double-edged sword” effect discussed by Webster et al. (2010) and Lepine et al. (2004), indicating that challenge stressors can result in both positive and negative outcomes. In contrast, hindrance stressors consistently exhibit detrimental effects, aligning with the findings of Cavanaugh et al. (2000) and Boswell et al. (2004). Hindrance stressors significantly increase the probability of knowledge hiding by reducing employees’ job crafting and increasing work withdrawal. Comparative group analysis further indicates that employees experiencing high levels of hindrance stressors exhibit lower job crafting, higher work withdrawal, and increased knowledge hiding, reinforcing the notion of a more pronounced positive impact of hindrance stressors on knowledge hiding.

The differential mechanisms of challenge stressors and hindrance stressors align with the fundamental principles of COR theory (Hobfoll et al., 2018). Specifically, challenge stressors stimulate job crafting in employees by providing resource returns, consistent with the resource-investment principle. However, individuals are more sensitive to resource loss than to resource acquisition, as suggested by the primacy-of-loss principle. Therefore, the level of work withdrawal triggered by challenge stressors exceeds job crafting, ultimately leading to increased knowledge hiding through the depletion path. The decrease in job crafting and the increase in work withdrawal caused by hindrance stressors, which only deplete personal resources, further support the primacy-of-loss principle.

#### 5.1.2 The moderating effect of empowering leadership

Empowering leadership plays a significant moderating role in the pathway through which hindrance stressors influence knowledge hiding via job crafting. As levels of empowering leadership increase, hindrance stressors lead to more knowledge-hiding behaviors through this pathway, highlighting the detrimental effects of excessive burdens associated with empowering leadership (Wong and Giessner, 2018). Nevertheless, empowering leadership does not show a significant moderating effect on the other three mediating paths, failing to support the “coping” and “buffering” hypotheses of the JD-R model (Lewig et al., 2007; Bakker et al., 2005). To clarify these findings, we conducted hierarchical regression analysis with empowering leadership as the independent variable (refer to Table 9). The results diverge from the poor performance of empowering leadership in its moderating role. Empowering leadership significantly negatively affects hindrance stressors (M4, *β* = −0.483, *p* < 0.01), work withdrawal (M8, *β* = −0.399, *p* < 0.01), and knowledge hiding (M10, *β* = −0.228, *p* < 0.01), while demonstrating a significant positive effect on job crafting (M6, *β* = 0.508, *p* < 0.01).

**Table 9:** Results of the hierarchical regression analysis (independent variable = empowering leadership).

	Challenge stressors		Hindrance stressors		Job crafting		Work withdrawal		Knowledge hiding	
	M1	M2	M3	M4	M5	M6	M7	M8	M9	M10
Control variables										
Gender	−0.208	−0.225	−0.292	−0.184	0.141	0.028	−0.423	−0.334	−0.442**	−0.391*
Age	0.113*	0.102	−0.071	0.001	0.164**	0.088**	−0.211**	−0.152*	−0.114*	−0.08
Education level	0.098	0.109*	0.157*	0.091	−0.061	0.009	0.192**	0.137*	0.126**	0.094*
Years of experience	0.032	0.031	−0.034	−0.028	0.03	0.024	−0.027	−0.023	0.006	0.008
Type of institution	−0.108	−0.11	−0.077	−0.065	0.027	0.014	−0.119	−0.11	−0.144**	−0.138*
Independent variable										
Empowering leadership		0.076		−0.483**		0.508**		−0.399**		−0.228**
*R* ^2^	0.047	0.053	0.03	0.164	0.097	0.528	0.071	0.151	0.077	0.143
Δ*R*^2^	0.037	0.041	0.02	0.154	0.088	0.523	0.062	0.141	0.068	0.132
*F*	4.830***	4.534***	2.997***	15.877***	10.502***	90.771***	7.470***	14.421***	8.130***	13.476***

*p < 0.05; **p < 0.01; ***p < 0.001.

Empowering leadership serves as a moderator in the pathway of “hindrance stressors → job crafting → knowledge hiding.” This finding confirms that excessive empowering leadership can overwhelm employees facing high levels of hindrance stressors (Magni and Maruping, 2013). Employees have already utilized personal resources to cope with these stressors. However, when high levels of empowering leadership demand additional extra-role behaviors, it exacerbates their resource depletion. As a result, employees may engage in reducing job crafting, potentially leading to a rise in knowledge-hiding behaviors.

The absence of a significant mediating role of empowering leadership across other pathways may be attributed to various reasons. Firstly, empowering leadership does not play a significant moderating role in the mediating pathway of “hinderance stressors → work withdrawal → knowledge hiding.” One possible reason for this is that hinderance stressors are often too overwhelming for employees to overcome or change (Cavanaugh et al., 2000). Regardless of whether leadership is empowering, the depletion of individual resources inevitably leads employees to engage in work withdrawal to conserve as many resources as possible. Secondly, empowering leadership does not exhibit a significant moderating effect in the two mediating pathways between challenge stressors and knowledge hiding. This is not the first instance of observing that leadership styles do not significantly moderate the relationship between challenge stressors and outcome variables in organizational contexts in China (Ding et al., 2017; Yao and Luo, 2022). It is important to note that the effectiveness of leadership depends on the quality of interaction and cooperation between leaders and their members (Uhl-Bien et al., 2014). Effective empowering leadership must be built on a solid foundation of robust leader-member relationships and mutual trust (Martin et al., 2016; Zhang, 2017). Additionally, the range of empowerment provided by leaders should align with the capabilities of their members (Cheong et al., 2016). By meeting these two conditions, empowering leadership can potentially exert its expected positive effects, helping employees better cope with challenge stressors and achieve favorable organizational outcomes. However, China is considered a traditional high power-distance country (Arrindell, 2003; Rui et al., 2023). In such context, employees typically accept the status differences between themselves and their leaders, as well as a work model that involves executing decisions made by their leaders (Lam and Xu, 2019). Consequently, employees are not adept at establishing an equitable exchange relationship with leaders (Farh et al., 2007). This cultural context presents a greater challenge for empowering leadership to have a positive impact within Chinese organizations.

### 5.2 Theoretical implications

This study validates the significant positive effects of challenge stressors and hindrance stressors on employees’ knowledge-hiding behavior. It addresses the ongoing debate in prior research about whether these two types of job stressors have positive or negative impacts on knowledge hiding. Additionally, it examines the consistency of these effects.

Furthermore, this study confirms the effectiveness of a newly developed parallel mediation model in explaining the relationship between job stressors and employees’ knowledge-hiding behavior. The discovery of two additional pathways, namely “job stressors → job crafting → knowledge hiding” and “job stressors → work withdrawal → knowledge hiding,” enriches the existing understanding of the mechanisms influencing knowledge-hiding behavior. The study also offers a plausible explanation for the stronger positive impact of hindrance stressors compared to challenge stressors. Hindrance stressors primarily deplete personal resources and can exacerbate knowledge-hiding behavior through a dual pathway: by reducing job crafting and increasing work withdrawal. Conversely, challenge stressors not only deplete personal resources, leading individuals to escalate work withdrawal, but they also provide resource rewards. This dynamic can promote job crafting and reduce the rate of knowledge-hiding behavior to some extent. These findings validate the CHSF’s binary distinction between challenge and hindrance stressors (Cavanaugh et al., 2000), the JD-R model’s dual-path hypothesis regarding motivation and depletion (Demerouti et al., 2001), as well as the principles of the COR theory related to primacy-of-loss and resource-investment (Hobfoll et al., 2018).

Lastly, this study highlights inconsistencies in the moderating effects of empowering leadership across various mediating pathways. It also examines its role as both an independent variable and as a moderating variable that influences job crafting, work withdrawal, and knowledge hiding. The results underscore the complex nature of empowering leadership. It can either serve as a valuable resource yielding positive outcomes or act as a burden that triggers negative consequences via different mechanisms (Cheong et al., 2016).

### 5.3 Practical implications

The findings of this study also offer practical implications for kindergarten administrators aiming to reduce preschool teachers’ knowledge-hiding behavior. Administrators should differentiate between challenge stressors and hindrance stressors for refined employee stress management. When dealing with challenge stressors, it is essential to manage them within a moderate range to enhance preschool teachers’ work motivation. This approach will promote job crafting and knowledge sharing among employees. If challenge stressors are too low, employees may resolve issues independently without feeling stimulated. Conversely, excessively high challenge stressors can overwhelm employees. This can lead to feelings of powerlessness and withdrawal from work, which may result in knowledge-hiding behaviors. The core principle of moderate challenge stressors involves setting realistic deadlines that convey a sense of urgency, as well as assigning challenging workloads and responsibilities. However, it is important to recognize that each teacher’s capacity to cope with challenge stressors varies. To appropriately set challenge stressors, it is crucial to consider each teacher’s teaching experience and professional background. This ensures that the tasks assigned are suitable for teachers at different stages of professional development. Additionally, comprehensive profiles for teachers should be developed. By consistently monitoring their performance, kindergarten administrators can gain a clearer understanding of each teacher’s capacity to handle challenge stressors. It is also vital to emphasize that any increase in challenge stressors should be accompanied by a corresponding increase in job resources (Lewig et al., 2007). Such resources include enhanced support from leadership and colleagues, timely feedback on performance, and greater autonomy in one’s work.

When it comes to hindrance stressors, the focus should be on minimizing them as much as possible. By reducing these stressors, administrators can alleviate preschool teachers’ concerns regarding resource loss. They also decrease work withdrawal and ultimately mitigate the likelihood of knowledge hiding. Kindergarten administrators should establish well-defined management systems and ensure their effective implementation in practice. This includes clarifying teachers’ responsibilities to reduce role ambiguity, streamlining workflows to eliminate bureaucratic obstacles, conducting fair performance evaluations to provide appropriate rewards, and safeguarding teachers’ rights to enhance job security. These strategies are expected to effectively lessen the hindrance stressors that preschool teachers may encounter (Zheng et al., 2020). By doing so, teachers can minimize the depletion of their time, energy, and emotional resources spent on managing these stressors.

Furthermore, kindergarten administrators should focus on enhancing their empowering skills to establish the necessary boundary conditions for empowering leadership to have a positive impact. Firstly, administrators should cultivate strong leader-member relationships and foster trust among teachers (Harris et al., 2014). This will enable teachers to establish better leader-member exchanges, resulting in their feeling strong support from their leaders and maintaining adequate resource status. Secondly, it is crucial for administrators to assess the levels of stressors that teachers face in a scientific manner. They should avoid excessive empowerment when employees are already heavily burdened by their workloads (Magni and Maruping, 2013). When employees experience high levels of job stressors, empowering leadership can become overwhelming rather than beneficial. Finally, administrators must recognize the importance of aligning their perceptions of empowerment with those of their members. They should strive to minimize potential misunderstandings among teachers regarding empowerment, particularly the idea that trivial tasks are assigned to them by leaders. When delegating responsibilities to preschool teachers, administrators must clearly articulate the objectives and significance of the tasks, convey their confidence in the teachers’ abilities, and specify the scope of autonomy granted. This approach encourages teachers to perceive empowering leadership as a recognition of their skills and contributions, ultimately enhancing the benefits of such leadership.

Additionally, it is recommended that kindergartens establish regular staff training programs and platforms for experience sharing, utilizing both face-to-face and online formats. This approach will help teachers continuously acquire knowledge and foster a culture of sharing and mutual assistance among them. If resources allow, material or spiritual rewards can also be offered to teachers who actively engage in knowledge-sharing activities.

### 5.4 Limitations and future research

While this study has made contributions to the field of knowledge hiding, there are several limitations that require discussion. Firstly, knowledge hiding is an adaptive coping behavior that manifests in specific situations, showing high variability among individuals. Therefore, we propose incorporating diary studies for follow-up investigations. Not only do they yield more accurate data, but they also shed light on how job stressors influence employees’ knowledge-hiding behavior over both short and long periods. Secondly, this study focused solely on preschool teachers in Jiangxi, Hubei, and Guangdong provinces in China, with female subjects accounting for over 97% of the participants. Although the gender distribution mirrors the actual scenario among preschool teachers in China, and gender was considered as a control variable in data analysis, it is recommended to include more diverse samples in future studies to enhance the generalizability of the findings. Thirdly, the factors contributing to employees’ knowledge-hiding behavior are multifaceted and involve intricate underlying mechanisms. Future research should consider incorporating new theories, such as affective events theory and the stimulus-organism-response model. Additionally, integrating new variables, such as personality traits and job characteristics, would allow for a more holistic examination. Furthermore, this study did not delve deeply into the impacts and mechanisms of empowering leadership on job crafting, work withdrawal, and knowledge hiding. Hence, future studies should not solely focus on empowering leadership but should also examine leader-member exchange. It is equally important to consider both administrators’ and employees’ perceptions of empowering leadership. This dual approach will enhance our understanding of the boundary conditions and underlying mechanisms through which empowering leadership operates.

### 5.5 Conclusion

Knowledge is an essential resource for individuals, and people exhibit heightened sensitivity toward it. The sharing of knowledge may be driven by a desire to acquire personal resources in return, while the hiding of knowledge may stem from a desire to avoid personal resource loss. Hindrance stressors play a significant role in driving knowledge-hiding behavior by limiting the potential for job crafting and increasing work withdrawal. Conversely, challenge stressors function as a double-edged sword. They encourage knowledge-hiding behavior by fostering work withdrawal but also discourage it by promoting job crafting. This results in a weaker overall impact. The dual effect underscores the intricate relationship between job stressors and knowledge-hiding behavior. Moreover, the dynamics of empowering leadership are complex and can lead to a range of outcomes, both beneficial and detrimental, under differing circumstances. The outcomes of this study support the idea that there is an increasing body of evidence suggesting that what is traditionally deemed a “good” factor may sometimes yield “bad” results (Ma and Tang, 2022). This is similar to the case of challenge stressors and empowering leadership in this research. Hence, it is crucial to recognize that a “good” factor may yield beneficial effects only under specific conditions. Therefore, further evidence is required to clarify the boundary conditions and the underlying mechanisms.

## Data Availability

The raw data supporting the conclusions of this article will be made available by the authors, without undue reservation.

## Footnotes

^1^ https://www.wjx.cn/
