# Supplementary material for: The impact of challenge and hindrance stressors on knowledge hiding: the mediating role of job crafting and work withdrawal
Source: Front Psychol. 2024 Nov 26;15:1465480. doi: 10.3389/fpsyg.2024.1465480 (PMC11628296; doi:10.3389/fpsyg.2024.1465480)
Supplement: Supplementary file 1 [file Presentation_1.pdf]

Supplementary Material

1 Measures of Challenge and Hindrance Stressors by Cavanaugh et al. (2000)

1. The number of projects and/or assignments I have
2. The amount of time I spend at work
3. The volume of work that must be accomplished in the allotted time
4. Time pressures I experience
5. The amount of responsibility I have
6. The scope of responsibility my position entails
7. The degree to which politics rather than performance affects organizational decisions
8. The inability to clearly understand what is expected of me on the job
9. The amount of red tape I need to go through to get my job done
10. The lack of job security I have
11. The degree to which my career seems "stalled'

Note: Items 1 to 6 reflect challenge stressors, and items 7 to 11 reflect Hindrance Stressors.

2 Measures of Knowledge Hiding by Connelly et al. (2012)

1. Agreed to help him/her but never really intended to
2. Agreed to help him/her but instead gave him/her information different from what s/he wanted
3. Told him/her that I would help him/her out later but stalled as much as possible
4. Offered him/her some other information instead of what he/she really wanted
5. Pretended that I did not know the information
6. Said that I did not know, even though 1 did
7. Pretended I did not know what s/he was talking about
8. Said that I was not very knowledgeable about the topic
9. Explained that I would like to tell him/her, but was not supposed to
10. Explained that the information is confidential and only available to people on a particular project
11. Told him/her that my boss would not let anyone share this knowledge
12. Said that 1 would not answer his/her questions

Note: Items 1 to 4 reflect evasive hiding, items 5 to 8 reflect playing dumb, and items 9 to 12 reflect rationalized hiding.

3 Measures of Job Crafting by Slemp & Vella-Brodrick (2013)

1. Introduce new approaches to improve your work
2. Change the scope or types of tasks that you complete at work
3. Introduce new work tasks that you think better suit your skills or interests
4. Choose to take on additional tasks at work
5. Give preference to work tasks that suit your skills or interests
6. Think about how your job gives your life purpose
7. Remind yourself about the significance your work has for the success of the organization

8. Remind yourself of the importance of your work for the broader community
9. Think about the ways in which your work positively impacts your life
10. Reflect on the role your job has for your overall well-being
11. Make an effort to get to know people well at work
12. Organize or attend work related social functions
13. Organize special events in the workplace (e.g., celebrating a co-worker's birthday)
14. Choose to mentor new employees (officially or unofficially)
15. Make friends with people at work who have similar skills or interests

Note: Items 1 to 5 reflect task crafting, items 6 to 10 reflect cognitive crafting, and items 11 to 15 reflect relational crafting.

4 Measures of Work Withdrawal by Lehman & Simpson (1992)

1. Thoughts of being absent
2. Chat with co-workers about nonwork topics
3. Left work station for unnecessary reasons
4. Daydreaming
5. Spent work time on personal matters
6. Put less effort into job than should have
7. Thoughts of leaving current job
8. Let others do your work
9. Left work early without permission
10. Taken longer lunch or rest break than allowed
11. Taken supplies or equipment without permission
12. Fallen asleep at work

Note: Items 1 to 8 reflect psychological withdrawal behaviors, and items 9 to 12 reflect physical withdrawal behaviors.

5 Measures of Empowering Leadership by Ahearne et al. (2005)

1. My manager helps me understand how my job fits into the bigger picture.
2. My manager helps me understand how my objectives and goals relate to that of the company.
3. My manager helps me understand the importance of my work to the overall effectiveness of the company.
4. My manager makes many decisions together with me.
5. My manager provides many opportunities for me to express my opinions.
6. My manager believes that I can handle demanding tasks.
7. My manager believes in my abilities to improve even when I make mistakes.
8. My manager makes it more efficient for me to do my job by keeping the rules and regulations simple.
9. My manager allows me to do my job my way.
10. My manager allows me to make important decisions quickly to satisfy customer needs.

Note: Items 1 to 3 reflect enhancing the meaningfulness of work, items 4 to 5 reflect fostering participation in decision making, items 6 to 7 reflect expressing confidence in high performance, and items 8 to 10 reflect providing autonomy from bureaucratic constraints.
